# Genome sequence of a symbiotic cyanobacterium from the flowering plant *Gunnera tinctoria*

**DOI:** 10.1128/MRA.00563-23

**Published:** 2023-10-16

**Authors:** Warren Shou Leong Ang, Olivia Burleigh, Sarah Frail, Yago V. S. Santos, Bhavyaa Tyagi, Fay-Wei Li

**Affiliations:** 1 Boyce Thompson Institute, Ithaca, New York, USA; 2 Department of Integrative Biology, Oregon State University, Corvallis, Oregon, USA; 3 Department of Biochemistry, Stanford University School of Medicine, Stanford, California, USA; 4 Department of Biology, University of New Mexico, Albuquerque, New Mexico, USA; 5 Department of Earth, Marine and Environmental Sciences, University of North Carolina, Chapel Hill, North Carolina, USA; 6 Plant Biology Section, Cornell University, Ithaca, New York, USA; The University of Arizona, Tucson, Arizona, USA

**Keywords:** cyanobacteria, symbiosis, *Gunnera*

## Abstract

Metagenomic analysis of the symbiotic cyanobacteria colonies within *Gunnera tinctoria* stems revealed a new strain of *Nostoc*. Here, we report its genome sequence.

## ANNOUNCEMENT

A few disparate land plant lineages had evolved symbiotic relationships with cyanobacteria to gain access to bioavailable nitrogen (1). *Gunnera* is the only flowering plant genus known to have cyanobacterial symbiosis and is of particular interest for agricultural engineering. However, very few symbiotic cyanobacterial genomes have been sequenced to date (2) and none from *Gunnera* spp.

Here, we generated a metagenomic data set from the cyanobacteria colonies inside the stem of *Gunnera tinctoria* (3), from which we extracted a near-complete *Nostoc* sp. genome. The *G. tinctoria* plants were originally purchased from Keeping It Green Nursery (Stanwood, WA). For DNA extraction, 1.6 g of stem sections containing cyanobacteria was dissected from a single adult plant. The tissue was washed with sterile water, and genomic DNA was extracted using DNeasy Plant Mini Kit (Qiagen, Germantown, MD). Purified DNA was concentrated using AMPure XP beads (Beckman Coulter, Indianapolis, IN) at 1× ratio, and no shearing was applied. A total of 0.75 µg of DNA was used for library preparation with the Ligation Sequencing Kit (SQK-LSK109; Oxford Nanopore Technologies, UK). The library was then loaded onto a MinION R9.4.1 Flow Cell (Oxford Nanopore Technologies, UK) and ran for 72 hours.

Default parameters were used for all software processing unless otherwise stated. After base calling using Guppy v6.4.2 in the super accuracy mode, a total of 6.5 Gb of raw reads were obtained, with an N50 length of 1,272 bp and a mean quality of 11.53. The reads were trimmed using PoreChop (v0.2.4 https://github.com/rrwick/Porechop), and those longer than 1 kilobase (kb) were used for metagenomic assembly in Flye v2.9.2 (4) with the “-meta” parameter enabled. The resulting assembly was polished by Medaka v1.8.1 (https://github.com/nanoporetech/medaka). To pull out the cyanobacterial genome, we classified the assembled contigs using Kraken2 v2.1.3 (5, 6) and extracted the contigs that were annotated as bacterial. Raw nanopore reads were then mapped to these bacterial contigs using minimap2-2.6 (r1175) (7, 8), and the mapped reads were subjected to a second round of metagenome assembly using Flye. This assembly was classified again using Kraken2. We kept contigs that were classified as cyanobacteria, were longer than 10 kb, and had a read depth between 25 and 100. The filtered contigs were polished by Medaka and corrected for frameshifts by Proovframe v0.9.7 (9).

Our final assembly consists of six contigs, totaling 7,907,695 bp. The largest contig was 7,292,919 bp, and two of the smaller contigs were circular plasmids of 221,999 bp and 361,828 bp. BUSCO (10) analysis showed 98% completeness when compared against the cyanobacteria database. The genome was then annotated using PGAP (11). The 16S rDNA sequences were BLAST against GenBank to identify closely related species. Of the two *Nostoc* species with the highest 16S identity, we calculated average nucleotide identity (ANI) using FastANI (12). This gave us a score of 88.1% and 87.7% when compared to *Nostoc* sp. ATCC 53789 (GenBank CP046703.1) and *Nostoc* sp. TCL240-02 (GenBank CP040094.1), respectively.

## Data Availability

The total raw nanopore reads were deposited under the BioProject PRJNA986849 (SRA accession: SRR25004942). The assembly is available at GenBank under the accession JAUDRJ000000000.
